# In-channel cryoprobe extraction for mediastinal and transbronchial cryobiopsy: a novel technique (cross-sectional study)

**DOI:** 10.1097/MS9.0000000000004039

**Published:** 2025-10-10

**Authors:** Miguel Angel Ariza-Prota, Javier Pérez-Pallarés, Lucía García Alfonso, Angela Lanza Martínez, Héctor Torres-Rivas, Luis Fernández-Fernández, María de la paz González Gutiérrez, Mario Berríos-Hernández, Marta García-Clemente, Francisco Julién López-González

**Affiliations:** a Division of Respiratory Medicine, Interventional Pulmonology Unit, Hospital Universitario Central de Asturias, Oviedo, Spain; b Division of Respiratory Medicine, Interventional Pulmonology Unit, HLA Hospital La Vega, Murcia, Spain; c Division of Pathology, Hospital Universitario Central de Asturias, Oviedo, Spain

**Keywords:** cryobiopsy, EBUS, mediastinal cryobiopsy, transbronchial cryobiopsy, working channel

## Abstract

**Background:**

Cryobiopsy has revolutionized mediastinal and lung parenchymal sampling, offering superior diagnostic yields compared to traditional techniques. However, the need for *en bloc* removal of the bronchoscope and cryoprobe during sample retrieval compromises the procedural safety and efficiency. We introduced a novel in-channel cryobiopsy extraction method that preserves continuous airway visualization and simplifies the procedural workflow.

**Methods:**

In this prospective study, we evaluated 30 cases of mediastinal cryobiopsy through the working channel guided by endobronchial ultrasound (C-Cryo-EBUS) and 10 cases of transbronchial cryobiopsy through the working channel (channel-guided transbronchial cryobiopsy (C-TBCB)], using 1.1 and 1.7 mm cryoprobes. After a standardized 3-s freeze, all tissue samples were extracted directly through the bronchoscope’s working channel without removing the scope. The outcomes assessed included procedural feasibility, diagnostic yield, procedure time, complication rate, and sample quality.

**Results:**

All cryobiopsies were successfully retrieved through the working channel, achieving a 100% diagnostic yield in both mediastinal and transbronchial settings. No complications or bronchoscopic damage was observed. Mean procedure times were 15.7 ± 6.3 min (C-Cryo-EBUS) and 14.6 ± 5.8 min (C-TBCB). Continuous visualization throughout the procedure enhanced control, minimized airway trauma, and enabled the immediate management of potential bleeding. The extracted specimens maintained excellent architecture and diagnostic adequacy, comparable to those of traditional methods, but with reduced procedural complexity.

**Conclusion:**

The in-channel cryoprobe extraction technique is a feasible, reproducible, and safe alternative to conventional cryobiopsy. It maintains endoscopic visualization, reduces procedure time, and preserves diagnostic sample quality. Its implementation may optimize clinical workflows and promote safer and more efficient cryobiopsy practices in interventional pulmonology.

## Introduction

Cryobiopsy is increasingly recognized as a valuable technique for sampling both mediastinal lymph nodes and lung parenchyma because of its superior tissue yield compared to transbronchial needle aspiration (TBNA) and conventional forceps biopsy^[1–4]^. While endobronchial ultrasound-guided transbronchial needle aspiration (EBUS-TBNA) remains the standard for mediastinal lymphadenopathy, its diagnostic accuracy may be limited in certain clinical scenarios, including lymphoma, granulomatous diseases, and necrotic nodes. In this context, mediastinal cryobiopsy (cryo-EBUS), an approach that integrates EBUS guidance with cryoprobe-based tissue acquisition, has emerged as a promising alternative, providing larger and more architecturally specimens^[5–7]^. Similarly, transbronchial lung cryobiopsy (TBCB) is increasingly utilized in the diagnostic work-up of diffuse interstitial lung diseases (ILDs), achieving definitive diagnoses when other modalities fail^[8,9]^.

**HIGHLIGHTS**

- In-channel cryoprobe extraction preserves continuous airway visualization.
- Achieves 100% diagnostic yield in mediastinal and transbronchial cryobiopsies.
- Mean procedure times: 15.7 ± 6.3 min (C-Cryo-EBUS) and 14.6 ± 5.8 min (channel-guided transbronchial cryobiopsy).
- No complications or bronchoscopic damage were observed.
- Specimens maintained excellent architecture and diagnostic adequacy.

Traditionally, both cryo-EBUS and TBCB require *en bloc* extraction of the bronchoscope and cryoprobe to retrieve frozen samples. Although effective, this approach results in the loss of airway visualization, prolongs procedural time, and may increase the risk of complications^[10,11]^. We present our experience with an innovative in-channel extraction technique that allows sample retrieval through the bronchoscope’s working channel without scope removal. This study aims to prospectively evaluate this novel approach by determining the procedural success rate of the in-channel extraction technique; assessing the diagnostic yield and quality of the samples obtained; evaluating the safety profile by monitoring for procedure-related complications; and measuring the procedure time to provide a preliminary comparison against times reported for conventional *en bloc* methods.

This cohort/cross-sectional/case-control study has been reported in line with the STROCSS guidelines[12].

## Methods

This prospective study was conducted between January and April 2025 to evaluate the diagnostic yield, feasibility, and safety of a novel in-channel cryoprobe extraction technique for both mediastinal and transbronchial cryobiopsies. To provide context for our novel technique, a targeted literature search of the PubMed/MEDLINE database was conducted, including articles published up to April 2025. The search strategy utilized keywords such as “mediastinal cryobiopsy,” “transbronchial cryobiopsy,” “EBUS,” “in-channel extraction,” and “interstitial lung disease.” We prioritized English-language systematic reviews, society guidelines, and seminal articles to build the foundation for the introduction and discussion sections. Channel-guided cryo-EBUS (C-Cryo-EBUS) is defined as mediastinal cryobiopsy performed through the working channel of the EBUS bronchoscope, without removal of the scope from the airway during specimen retrieval. Similarly, channel-guided transbronchial cryobiopsy (C-TBCB) refers to transbronchial cryobiopsy conducted through the working channel of the bronchoscope without scope removal during sample extraction. Forty consecutive patients were enrolled, of whom 30 underwent C-Cryo-EBUS and 10 underwent C-TBCB. The primary outcome was procedural feasibility, defined as successful retrieval of the biopsy sample through the working channel. Secondary outcomes included diagnostic yield, procedure time, complication rates (including bleeding, pneumothorax, and bronchoscopic damage), and histopathological sample quality. Diagnostic yield was defined as the percentage of patients in whom the biopsy specimens led to a specific histopathological diagnosis, as determined by the pathology report. Post-procedural chest radiographs were obtained in all patients to assess pneumothorax or pneumomediastinum. Pathologists evaluating the samples were blinded to the extraction techniques used. For C-TBCB samples, two pathologists independently assessed the specimens and subsequently reached a consensus on all diagnoses, ensuring a robust final diagnosis. A formal statistical measure of inter-rater reliability was not calculated.

### Complication monitoring and follow-up

Complications were prospectively monitored and defined as follows:

Bleeding: Defined as any hemorrhage requiring intervention (e.g., suctioning beyond simple clearance, administration of cold saline, or balloon tamponade). Bleeding was collected into a mucus reservoir (Mocstrap; Proclinics, Barcelona, and Spain), which has a scale in milliliters, and quantified according to the following classification: mild haemorrhage, bleeding <10 mL; moderate, 10–40 mL; severe, >40 mL. Minor, self-limiting bleeding was not classified as a complication.

Pneumothorax/pneumomediastinum: Assessed via post-procedural chest radiography in all patients. Any detectable air leak was considered a complication.

Bronchoscopic damage: The integrity of the bronchoscopes was systematically assessed through a two-step process. First, following every procedure, no technical incidents were recorded, and all endoscopes successfully passed standard post-procedural leak testing to ensure their integrity. Second, to further rule out any latent damage, both the therapeutic bronchoscope (Pentax EB-1975 K with a 2.8 mm working channel) and the echobronchoscope (Pentax EB-1970UK) underwent a formal maintenance review by the manufacturer’s authorized service center upon completion of the study. This comprehensive technical inspection confirmed the absence of any signs of damage within the working channels of the scopes.

Follow-up was conducted via telephone at 24 h for all patients. For the C-TBCB group, this 24-h follow-up was the final point of contact, consistent with standard protocols for monitoring acute complications like pneumothorax. For the C-Cryo-EBUS group, an additional follow-up was performed at 2 weeks to assess for any delayed adverse events.

### C-Cryo-EBUS technique

C-Cryo-EBUS procedures were performed using a linear echobronchoscope (Pentax EB-1970UK, J10 series), 22-gauge needle (SonoTip TopGain, Medi-Globe, Germany), and 1.1-mm cryoprobe (Erbecryo 20402-401, Erbe Elektromedizin GmbH, Tübingen, Germany). For C-Cryo-EBUS, any patient with mediastinal or hilar lymphadenopathy identified on prior imaging that was accessible via EBUS was considered for inclusion. There were no specific pre-selection criteria based on lymph node size. The technique was based on the Ariza-Pallarés method for tunneling prior to cryobiopsy extraction[10]. Following identification of the target lymph node station under EBUS guidance, one to two conventional TBNAs were performed. Because the procedure was ultrasound-guided, it was crucial to visualize the TBNA puncture tract within the lymph node. After completion of TBNA sampling, a 1.1-mm cryoprobe was introduced through the working channel of the echobronchoscope. Under continuous EBUS visualization, the cryoprobe was advanced toward the puncture site and gently inserted through the established tract. Once positioned at the target site, the cryoprobe was stabilized using the operator’s first and second fingers at the entry point of the bronchoscope’s working channel. Cryoactivation was performed by cooling with liquid carbon dioxide for 3 s. The cryoprobe, with the frozen tissue attached, was carefully retracted through the working channel while the pedal was maintained until the sample was secured outside the airway. Three cryobiopsies were performed for each lymph node. All specimens were immediately placed in a saline solution and fixed in formalin. Postprocedural chest radiography was performed in all the patients to rule out pneumothorax. Patients were discharged after 1 h of observation if no complications were noted. Follow-up was conducted 24 h via telephone and again at 2 weeks post-procedure to assess for any delayed adverse events.

### C-TBCB technique

All patients were referred to the interventional pulmonology unit after evaluation by the multidisciplinary committee on diffuse ILDs. Patients were enrolled if they demonstrated clinical and radiographic features of ILD on high-resolution computed tomography (HRCT) that were inconsistent with the usual interstitial pneumonitis (UIP) pattern. Patients exhibiting HRCT findings typical of UIP were excluded from the study. TBCBs were performed in the most radiographically affected lobes, as determined by chest imaging. Two pathologists independently assessed the tissue specimens and reached a consensus on the histopathological diagnoses. Tissue sample diameter and procedure duration were recorded for each patient. Patients were excluded if they had blood dyscrasias, severe respiratory failure, unstable cardiac conditions, pulmonary hypertension, received anticoagulant therapy, or had a diffusing capacity of the lung for carbon monoxide of less than 30%.

All C-TBCB procedures were performed under general anesthesia with endotracheal intubation. Procedures were conducted using a therapeutic bronchoscope (Pentax EB-1975K, with a 2.8 mm working channel diameter), a 1.7 mm cryoprobe (Erbe, Germany), and a bronchial blocker (Arndt, Cook Medical, USA). The cryoprobe diameters were selected to maximize sample size while ensuring compatibility with the working channels of the respective bronchoscopes. The 1.1 mm cryoprobe was used for the standard EBUS scope (Pentax EB-1970UK), while the larger 1.7 mm probe was used with the therapeutic bronchoscope (Pentax EB-1975K), which features a wider 2.8 mm working channel, for the C-TBCB procedure. A fluoroscopy was not performed. After inserting the bronchoscope with the occlusion balloon attached, the target segment was identified, and the balloon was deployed to confirm proper positioning and function within the intended lobe or segment. The 1.7 mm cryoprobe, connected to a cryotherapy system (Erbokryo Ca, Erbe, Germany), was then advanced through the working channel of the bronchoscope until gentle resistance was encountered, indicating the distal advancement limit. The probe was subsequently retracted by approximately 1–2 cm to obtain the optimal sampling position. With the probe positioned appropriately, the operator stabilized it by firmly securing the cryoprobe near the working channel entry by using the thumb and index finger. The freezing pedal was then activated for 3 s, and immediately after, the cryoprobe with the attached cryobiopsy specimen was extracted through the working channel while the pedal was maintained until the sample was completely externalized from the airway. Subsequently, the specimens were thawed and processed for histopathological analysis. Postprocedural chest radiography or pleural ultrasound was performed to rule out pneumothorax. The patients were discharged 2 h after confirming the absence of immediate complications. All patients underwent follow-up evaluation at 24 h via telephone to assess any delayed complications.

**Figure 1.**
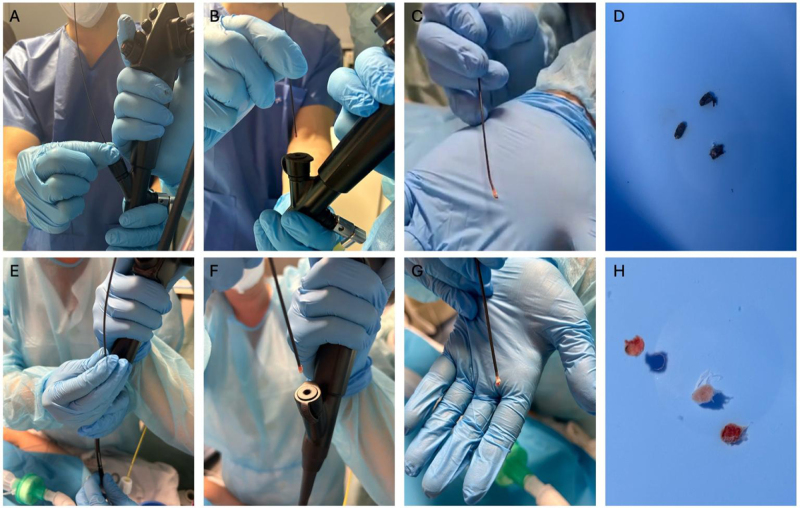
Cryobiopsy through the working channel guided by EBUS (C-Cryo-EBUS): Step-by-Step. (A) Once the 1.1 mm cryoprobe is positioned at the desired site for mediastinal cryobiopsy, the probe is firmly held with the thumb and index finger of the dominant hand, as close as possible to the exit of the working channel, using the nondominant hand for support and stabilization. (B) The pedal is activated for 3 s, followed by an immediate, firm, and controlled upward pull directed toward the ceiling to extract the sample (pedal pressed until the sample is outside the EBUS scope). (C) Cryobiopsy specimen immediately after extraction. (D) Three mediastinal cryobiopsy samples. Transbronquial cryobiopsy through the working channel (C-TBCB): Step-by-Step. (E) Once the 1.7 mm cryoprobe is positioned at the desired site for transbronchial cryobiopsy, it is held in a similar manner, using the thumb and index finger of the dominant hand near the working channel outlet. (F) The pedal is activated for 3 s, followed by a controlled upward pull (pedal pressed until the sample is outside the EBUS scope). (G) Cryobiopsy specimen adhered to the cryoprobe. (H) Three transbronchial cryobiopsy samples.

Both procedures were consistent and reproducible (Fig. 1). Additionally, Supplemental Digital Content Video S1, available at http://links.lww.com/MS9/A988 demonstrates the step-by-step extraction of the cryoprobe through the working channel during C-Cryo-EBUS, whereas Supplemental Digital Content Video S2, available at http://links.lww.com/MS9/A989 shows the same in-channel extraction technique applied during C-TBCB. These complementary videos aimed to visually reinforce the feasibility and reproducibility of this novel approach in both mediastinal and transbronchial cryobiopsy procedures.

Data were analyzed using Stata v15.1 software (StataCorp, 2015). Qualitative variables were assessed using the *χ*^2^ test, while quantitative variables were analyzed using the Student’s *t*-test or analysis of variance. Results are presented as means with standard deviations in parentheses.

## Results

A total of 30 C-Cryo-EBUS and 10 C-TBCB procedures were performed using the in-channel extraction technique. The mean patient age was 64.7 years in the C-Cryo-EBUS group and 65.8 years in the C-TBCB group, with male predominance in both groups (70 and 80%, respectively). All cryobiopsy samples were successfully extracted through the working channel, yielding intact and diagnostic samples and resulting in a 100% diagnostic yield in both groups. On an average, three cryobiopsies were obtained for each patient. The most frequently sampled mediastinal lymph node stations were stations 7 and 11L (*n* =6 each), followed by station 4R (*n* = 4). For TBCB, the right lower lobe was the most sampled (*n* = 5).

C-Cryo-EBUS procedures were performed under moderate conscious sedation using midazolam and fentanyl with oral access through a short bite block. In contrast, C-TBCB was administered under general anesthesia with endotracheal intubation. No procedural complications or bronchoscopic damage were observed in either group. The mean procedure duration was 15.7 ± 6.3 min for C-Cryo-EBUS and 14.6 ± 5.8 min for C-TBCB. The average diameter of the obtained tissue samples was 0.48 and 0.67 cm, respectively. All patients underwent postprocedural chest radiography, and no cases of pneumothorax or pneumomediastinum were identified. A detailed summary of the procedural characteristics and diagnostic outcomes of both the techniques is presented in Table 1.

**Table 1:** Summary of C-Cryo-EBUS and C-TBCB procedures

	C-Cryo-EBUS	C-TBCB
Number of patients	30	10
Mean age (years)	64.7	65.8
Male/female (%)	70/30	80/20
Sample obtained (%)	100%	100%
Representative sample (%)	100%	100%
Diagnostic yield (%)	100%	100%
Mean number of biopsies	3	3
Most common site	Station 7	RLL
Procedure time (min)	15.7 ± 6.3 (6–28.2)	14.6 ± 5.8 (7–25.8)
Mean sample diameter (cm)	0.48	0.67
Complications	None	None
Bronchoscope damage	None	None
Diagnoses (*n*)	Lung adenocarcinoma (13)	UIP (2)
	Sarcoidosis (4)	Sarcoidosis (2)
	Squamous cell lung carcinoma (3)	Hypersensitivity pneumonitis (2)
	Negative for malignancy (3)	IPF (1)
	Anthracosis (3)	Asbestosis (1)
	Renal cell carcinoma (1)	Silicosis (1)
	Hodgkin lymphoma (1)	Eosinophilic pneumonia (1)
	Tuberculosis (1)	
	B cell lymphoma (1)	

Data are presented as the mean (range) or mean ± SD. C-Cryo-EBUS, channel cryobiopsy guided by endobronchial ultrasound; C-TBCB, channel transbronchial cryobiopsy; UIP, usual interstitial pneumonia; IPF, idiopathic pulmonary fibrosis; RLL; right lower lobe.

## Discussion

The in-channel extraction technique represents a significant advancement in the cryobiopsy methodology. By maintaining continuous endoscopic visualization, it overcomes one of the key limitations of the traditional *en bloc* extraction approach^[13,14]^. This is particularly beneficial in cryo-EBUS, where visual access to the target lymph node station allows for safe and efficient acquisition of multiple biopsies. During TBCB, continuous airway visualization enhances procedural control and facilitates early detection and prompt management of complications such as bleeding. A particularly notable advantage of transbronchial procedures is their ability to monitor bronchial blockers in real time. Direct visualization of balloon positioning and inflation helps to prevent unrecognized dislodgement or suboptimal segmental occlusion. This level of precision reduces the risk of uncontrolled hemorrhage, which is a critical safety concern for advanced bronchoscopic interventions.

In 2017, Lentz *et al* published a state-of-the-art review on transbronchial cryobiopsy for diffuse parenchymal lung disease. In their article, they describe the traditional technique for performing TBCB and include an image captioned: “Samples frozen by the cryoprobe are much too large to be withdrawn through the working channel, requiring the bronchoscope, cryoprobe, and frozen adherent specimen to be removed from the airway *en-bloc*.” In this figure, a 1.9 mm cryoprobe is frozen for 5 s in saline, generating a visible ice ball at the probe’s tip[15]. One of the reasons we believe that the cryoprobe was successfully withdrawn through the working channel during transbronchial cryobiopsy in our study is the nature of the biopsied tissue itself. Unlike a rigid ice ball, the tissue is soft and pliable. When extracted through the working channel, the specimen that adhered to the cryoprobe tended to elongate and adapt to the shape of the channel, facilitating a smooth passage. In fact, samples appear more elongated when attached to the tip of the probe and tend to return to their original rounded shape when placed in saline or formalin for processing. An additional consideration that may limit the generalizability of the authors’ conclusion is the use of an earlier-generation 1.9 mm cryoprobe, which has a wider diameter than the currently available 1.7 mm models. Furthermore, their methodology involved a 5-s freeze time. In contrast, our protocol employed a 3-s freeze, which, as demonstrated in our series, did not compromise diagnostic accuracy. Both the smaller probe diameter and shorter freeze duration are likely to improve the feasibility of in-channel extraction and may account for the differing outcomes between techniques.

Our findings also demonstrate a reduction in overall procedural time compared to a previously reported series employing the conventional *en bloc* extraction approach^[3,16]^. While a direct statistical comparison is not possible without a control group, this observation suggests our technique may improve procedural efficiency by eliminating the time-consuming step of bronchoscope re-insertion. By maintaining the bronchoscope *in situ*, we avoided repeated repositioning, thereby simplifying the procedure and potentially minimizing sedation or anesthesia requirements. Importantly, this new approach did not compromise the sample quality; all specimens were both diagnostic and representative. Pathologists were blinded to the procedural technique used to ensure unbiased assessment of sample adequacy and diagnostic interpretation. No procedural complications or bronchoscopic damage was observed. The primary risk mitigation strategy is the maintenance of continuous airway visualization, which allows for real-time monitoring of the biopsy site for immediate detection and management of bleeding. For C-TBCB, the use of a bronchial blocker, also under direct visual control, provides an additional layer of safety against significant hemorrhage.

Beyond the immediate clinical benefits, our in-channel extraction technique may serve as a foundational technology for the integration of artificial intelligence (AI) into cryobiopsy procedures. Recent breakthroughs have demonstrated AI’s transformative potential across medicine, from molecular biology with models like AlphaFold to image-based genomic prediction for tailored cancer therapy^[17,18]^. A key barrier to applying AI in real-time bronchoscopy has been the fragmented data streams from traditional techniques, particularly the loss of visualization during *en bloc* sample retrieval.

Our method overcomes this by providing a continuous, uninterrupted video feed of the entire biopsy process. This stable data stream is precisely the prerequisite needed to train and validate AI models for tasks such as automated anatomical landmark recognition, real-time tool navigation, and early complication alerts (e.g., detecting subtle bleeding). By creating a standardized, visualized workflow, this technique transforms cryobiopsy from a simple diagnostic tool into a rich data acquisition platform. In the future, this could enable the development of intelligent cryobiopsy systems, where AI could assist in guiding the probe to optimal sampling locations or even connect with predictive models to create a closed-loop diagnostic-to-therapeutic pathway. Thus, our technique not only refines current practice but also provides the necessary clinical interface for the next generation of human-machine collaborative diagnostics.

The primary limitations of our study include its single-center design and a relatively small sample size of 40 patients. Although our results are consistent and promising, they require validation in larger, multicenter trials to ensure generalizability. Our study also did not systematically collect detailed data on patient comorbidities or specific sedation dosages, which should be included in future, larger prospective trials to allow for more granular analysis. A potential challenge to the widespread adoption of this technique is the variability in working channel diameters, angulation capabilities, and flexibility across different bronchoscope models and manufacturers. Future studies should systematically evaluate the technique’s performance with a range of endoscopes to establish clear compatibility guidelines. Furthermore, a formal cost-effectiveness analysis is warranted. The potential for reduced procedure times, decreased anesthesia requirements, and minimized bronchoscope wear-and-tear suggests that this technique could lead to significant cost savings, though this requires dedicated economic study.

The in-channel extraction technique can potentially redefine cryobiopsy procedures in interventional pulmonology. By offering a safer, faster, and technically more accessible approach for obtaining high-quality tissue samples, broader implementation is supported. The associated reduction in anesthesia duration and procedural complexity may contribute to decreased resource utilization and enhanced patient outcomes. Furthermore, its ability to shorten the learning curve could facilitate the wider adoption of advanced diagnostic bronchoscopy techniques when high-fidelity tissue acquisition is vital for precision medicine.

## Conclusion

The in-channel cryobiopsy extraction technique, which preserves continuous endoscopic visualization while reducing the procedure time and maintaining diagnostic yield, has emerged as a promising advancement in both mediastinal and parenchymal sampling. Its technical simplicity and safety profile suggest its potential for widespread adoption, even in less-experienced settings. Further prospective multicenter studies are needed to validate these findings, assess long-term clinical outcomes, and confirm reproducibility across a variety of bronchoscopic platforms. Such evidence is essential to support generalizability and guide future efforts toward technical standardization.

## Data Availability

The datasets generated and/or analyzed during the current study are available from the corresponding author upon reasonable request.

## Research registration unique identifying number (UIN)

Research Registry https://www.researchregistry.com/browse-the-registry#home/. Identifying number is: researchregistry11232.
